# Noncoding variation of the gene for ferritin light chain in hereditary and age-related cataract

**Published:** 2013-04-11

**Authors:** Thomas M. Bennett, Giovanni Maraini, Chongfei Jin, Wenmin Sun, J. Fielding Hejtmancik, Alan Shiels

**Affiliations:** 1 Department of Ophthalmology and Visual Sciences, Washington University School of Medicine, St. Louis, MO; 2 Department of Ophthalmology, University of Parma, Parma, Italy; 3 Ophthalmic Genetics and Visual Function Branch, National Eye Institute, National Institutes of Health, Bethesda, MD

## Abstract

**Purpose:**

Cataract is a clinically and genetically heterogeneous disorder of the ocular lens and an important cause of visual impairment. The aim of this study was to map and identify the gene underlying autosomal dominant cataract segregating in a four-generation family, determine the lens expression profile of the identified gene, and test for its association with age-related cataract in a case-control cohort.

**Methods:**

Genomic DNA was prepared from blood leukocytes, and genotyping was performed by means of single-nucleotide polymorphism markers and microsatellite markers. Linkage analyses were performed using the GeneHunter and MLINK programs, and mutation detection was achieved by dideoxy cycle sequencing. Lens expression studies were performed using reverse-transcription polymerase chain reaction (RT–PCR) and in situ hybridization.

**Results:**

Genome-wide linkage analysis with single nucleotide polymorphism markers in the family identified a likely disease-haplotype interval on chromosome 19q (rs888861-[~17Mb]-rs8111640) that encompassed the microsatellite marker D19S879 (logarithm of the odds score [Z]=2.03, recombination distance [θ]=0). Mutation profiling of positional-candidate genes detected a heterozygous, noncoding G-to-T transversion (c.-168G>T) located in the iron response element (IRE) of the gene coding for ferritin light chain (*FTL*) that cosegregated with cataract in the family. Serum ferritin levels were found to be abnormally elevated (~fourfold), without evidence of iron overload, in an affected family member; this was consistent with a diagnosis of hereditary hyperferritinemia-cataract syndrome. No sequence variations located within the IRE were detected in a cohort of 197 cases with age-related cataract and 102 controls with clear lenses. Expression studies of human FTL, and its mouse counterpart FTL1, in the lens detected RT–PCR amplicons containing full-length protein-coding regions, and strong in situ localization of FTL1 transcripts to the lens equatorial epithelium and peripheral cortex.

**Conclusions:**

The data are consistent with robust transcription of FTL in the lens, and suggest that whereas variations clustered in the IRE of the FTL gene are directly associated with hereditary hyperferritinemia-cataract syndrome, such IRE variations are unlikely to play a significant role in the genetic etiology of age-related cataract.

## Introduction

Hereditary forms of cataract constitute a clinically and genetically heterogeneous disorder of the ocular lens (OMIM). They are usually diagnosed at birth (congenital), during infancy, or during childhood into adolescence, and despite surgical treatment, are an important risk factor for lifelong visual impairment [1-3]. In addition to being associated with many genetic syndromes and metabolic disorders involving other ocular and/or systemic abnormalities (OMIM), cataract may be inherited as an isolated or primary lens phenotype, often with autosomal dominant transmission and high penetrance [4-6]. Currently, mutations in at least 22 genes have been linked with autosomal dominant and recessive forms of primary cataract that variably affect all or specific regions of the lens (e.g., nuclear, lamellar, sutural, polar) and exhibit wide variation in size, shape, density, and color of opacity, making accurate phenotype-genotype correlations challenging [7]. The majority of underlying mutations have been detected in 12 genes encoding alpha-, beta- and gamma-crystallins, and connexins alpha-3 and alpha-8, with the remainder of mutations found in a functionally diverse group of genes including those for an aquaporin, a heat-shock transcription factor, and intermediate filament-like proteins [4-6]. Interestingly, there is increasing evidence that variations in several of the genes underlying rare Mendelian forms of cataract are also associated with much more frequent forms of age-related cataract [6]. For example, coding and noncoding variations in the genes for galactokinase deficiency (*GALK1*) and an ephrin receptor (*EPHA2*) have been associated with age-related cataract in divergent populations [8-12] providing new insights into the genetic complexity of this universally important cause of low vision and blindness. Here, we have mapped autosomal dominant cataract segregating in a Caucasian-American family to chromosome 19q, identified a recurrent mutation in the iron response element (IRE) of the gene for ferritin light chain or L-ferritin (*FTL*), localized expression of FTL in the lens, and evaluated sequence variation of the FTL-IRE region in a case-control cohort of age-related cataract.

## Methods

### Family participants

A four-generation Caucasian-American pedigree from the Midwestern United States was ascertained through ophthalmic records in the Department of Ophthalmology and Visual Sciences at Washington University School of Medicine. Blood samples were obtained from 13 family members, including six affected, four unaffected, and three spouses. Leukocyte genomic DNA was purified using the Gentra Puregene Blood kit (Qiagen, Valencia, CA), and quantified by absorbance at 260 nm (NanoDrop 2000, Wilmington, DE). Ethical approval for this study was obtained from the Washington University Human Research Protection Office, and written informed consent was provided by all participants before enrollment in accordance with the tenets of the Declaration of Helsinki, as well as Health Insurance Portability and Accountability Act regulations.

### Case-control participants

A case-control cohort of unrelated individuals aged >55 years from Northern Italy was ascertained from the Clinical Trial of Nutritional Supplements and Age-Related Cataract Study [13]. Genomic DNA was purified from blood samples using a standardized protocol that included detergent lysis of cells and high salt precipitation of proteins followed by ethanol precipitation and phenol/chloroform extraction of DNA as described [14]. Cataract status (nuclear, cortical, posterior subcapsular, clear lens) was evaluated by grading slit-lamp and retroillumination lens photographs according to a modification of the Age-Related Eye Disease Study cataract grading system as described [15]. Briefly, a lens was defined as clinically cataractous when at least one type of opacity was present with the following grades: nuclear opalescence >4.5 (range 0.9-8.1); cortical opacity >25% inside the 5 mm diameter circle at the center of the pupil and/or >25% outside the central 5 mm diameter circle; posterior sub-capsular opacity >12.5% inside the central 5 mm diameter circle. A clear lens was defined by the following grades: nuclear opalescence <3.0; cortical opacity <5% within the central 5 mm diameter circle, and <15% outside the central 5 mm diameter circle; posterior sub-capsular opacity <1.0% within the central 5 mm diameter circle. Ethical approval for this study was obtained from the University of Parma and the National Eye Institute, and written informed consent was provided in accordance with the tenets of the Declaration of Helsinki.

### Linkage analysis

For genome-wide linkage analysis, single-nucleotide polymorphism (SNP) genotyping was performed by means of the GeneChip Human Mapping 10 K Array Xba 142 2.0 (Affymetrix, Santa Clara, CA), in the GeneChip Core Facility at Washington University Siteman Cancer Center. Parametric multipoint linkage analysis was performed using an autosomal dominant model with GeneHunter Plus from the easyLINKAGE Plus (v. 5.08) package [16]. SNP marker allele frequencies used for linkage analysis were those calculated for Caucasians by the HapMap project. A frequency of 0.0001 and a penetrance of 100% were assumed for the disease allele. Computation was performed with GeneHunter Plus (v.1.2) in sets of 100 markers using the AFFY 10 K deCODE human genetic marker map. Confirmation of linkage was achieved by genotyping microsatellite markers from the NCBI combined Généthon, Marshfield, and deCODE genetic linkage maps, as described [17]. Pedigree and haplotype data were managed using Cyrillic (v. 2.1) software (FamilyGenetix Ltd., Reading, UK), and two-point logarithm of the odds (LOD) scores (Z) calculated using the MLINK sub-program from the LINKAGE (5.1) package of programs [18]. Marker allele frequencies were assumed to be equal, and a frequency of 0.0001 with a penetrance of 100% was assumed for the disease allele.

### Sequencing

Genomic sequence for lens intrinsic membrane protein-2 (Gene ID: 3982) and *FTL* (Gene ID: 2512) were obtained from the Ensembl human genome browser, and gene-specific M13-tailed PCR primers (Table 1) were selected from the NCBI resequencing amplicon probe database or custom designed with Primer Quest (IDT.com) or Exon Primer (UCSC Genome Bioinformatics). Exons were PCR amplified then cycle-sequenced in both directions using BigDye Terminator Mix (v3.1) and a 3130×l-16 Genetic Analyzer (Applied Biosystems, Foster City, CA), as described [9].

**Table 1:** PCR primers used for amplification of human FTL and LIM2, and mouse FTL1.

Primer	Location	Strand	Sequence (5′ - 3′)
FTL-IREF	5′-UTR	sense	ATTTCACAACACGCTGGCGCTACA
FTL-Ex1R	Intron-1	antisense	TCTGTTTACCCGACCGCACAAAGA
32G>TR	IRE	antisense	GTTCCGTCCAAACACTGTTGAAGA
FTL-Ex2F	Intron-1	sense	GAGTCCCCTTGGCCTCG
FTL-Ex2R	Intron-2	antisense	GACACCTACGCCCTCAAATC
FTL-Ex3F	Intron-2	sense	CCAACGACTCTTGGGAAATG
FTL-Ex3R	Intron-3	antisense	AAAGGGAGCAGAGGCTTGAG
FTL-Ex4F	Intron-3	sense	TCAGAGCCTCATTTCACACC
FTL-Ex4R	Exon-4	antisense	CCAACTCCTCTTTCACTGGC
FTL-Start	Exon-1	sense	ATGAGCTCCCAGATTCGTCAG
FTL-Stop	Exon-4	antisense	TTAGTCGTGCTTGAGAGTGAG
FTL1-Start	Exon-1	sense	ATGACCTCTCAGATTCGTCAG
FTL1-Stop	Exon-4	antisense	CTAGTCGTGCTTGAGAGTGAG
LIM2-Ex1F	Exon-1	sense	GGACTTCCAGGTTCTGAGCAAGG
LIM2-Ex1R	Intron-1	antisense	CGGACAAAGCCAGGGTTGCT
LIM2-Ex2F	Intron-1	sense	TGTGCATGACACCTCTGAAGCG
LIM2-Ex2R	Intron-2	antisense	GCATCCCACTCCTGAGCCCT
LIM2-Ex3F	Intron-2	sense	GCTGAGGTGGAAGCAGTCTTGC
LIM2-Ex3R	Intron-3	antisense	TTGCCAGGTGAAGAAGAGGGC
LIM2-Ex4F	Intron-3	sense	CCCAACACCCTACTCTCTTTCTCCC
LIM2-Ex4R	Intron-4	antisense	CCAAATATTGTTTCTCCCTCCCAGG
LIM2-Ex5F	Intron-4	sense	CCACCCTTACAGCTGTTTCTCCC
LIM2-Ex5R	Exon-5	antisense	CCCACATGAGTCCCACAGCA

Gene-specific primer-pairs used for amplification and sequencing of exons or transcripts.

### Serum ferritin and plasma iron profile

Fasting blood samples were collected by venipuncture into either anticoagulant-free tubes for serum, or lithium heparin tubes for plasma (BD, Franklin Lakes, NJ). Serum ferritin was measured by means of chemiluminescence enzyme immunoassay (ADVIA Centaur; Bayer, Pittsburg, PA). Plasma iron profile, including iron, iron-binding capacity, and transferrin saturation, was determined by colorimetric (Ferrochrome/Ferrozine) assay (Roche Diagnostics, Indianapolis, IN). Transferrin saturation (%) was calculated from iron concentration divided by total iron binding capacity. Assays were conducted according to Clinical Laboratory Improvement Amendments regulations at Barnes-Jewish Hospital Laboratory.

### Eye tissue collection and preparation

Mice (C57B6/J, postnatal day 21-28) were humanely killed by CO_2_ asphyxiation followed by cervical dislocation. Eyes were removed and fixed in 10% neutral buffered formalin (Fisher Scientific, Fair Lawn, NJ) for 24 h at 20 °C before histology using standard formaldehyde-fixed-paraffin-embedded (FFPE) techniques. Alternatively, mouse eyes were placed in pre-warmed (37°C) phosphate buffered saline (PBS; 10 mM phosphate buffer, 2.7 mM potassium chloride, 137 mM sodium chloride, pH 7.4) and lenses dissected through a posterior incision in the globe then stored (−20 °C) in RNAlater (Invitrogen, Carlsbad, CA). Postmortem human lenses were obtained (frozen on dry ice) from the Lions Eye Bank of Oregon. All tissue procurement procedures were approved by the Washington University Human Research Protection Office and Animal Studies Committee, and conformed to the guidelines published by the Institute for Laboratory Animal Research.

### Reverse-transcription polymerase chain reaction

Total cellular RNA was extracted from mouse and human lenses by means of the RNeasy Plus Micro kit (Qiagen) and TRIzol reagent (Invitrogen), respectively, then quantified by ultraviolet absorbance (ND2000, Nanodrop). Lens RNA (250 ng) was reverse transcribed in the presence of random hexamers with the iScript cDNA synthesis kit (Bio-Rad, Hercules, CA), and cDNA products amplified (GeneAmp 9700 thermal cycler, Applied Biosystems) with gene-specific primers (Table 1) using Top-Taq reagents (Qiagen) according to the manufacturer’s instructions. Reverse-transcription polymerase chain reaction (RT–PCR) amplicons were visualized (302 nm) by electrophoresis on 2% agarose-gels stained with GelRed (Biotium, Hayward, CA). Amplicon identity was confirmed by sequencing as described above.

### In situ hybridization

In situ hybridization (ISH) was performed using the RNAscope 2.0 FFPE Reagent Kit - RED (Advanced Cell Diagnostics, Inc. Hayward, CA) with custom synthesized target probes designed for the mouse FTL1 transcript (NM_010240.2, 986 bp mRNA), essentially as described [19]. The target probe region (6–908 bp) covered 5′-untranslated region (5′-UTR; 6–255 bp), coding sequence (256–807 bp), and 3′-UTR (808–908 bp). Briefly, FFPE microtome sections (5 µm, RM2255, Leica Microsystems, Buffalo Grove, IL) on glass slides (SuperFrost Plus) were baked (1 h, 60 °C), dewaxed in xylene, dehydrated in ethanol, boiled in citrate buffer, then protease treated (10 ug/ml) in a HybEZ Oven (40 °C, 30 min). Pretreated sections were hybridized with target probes (2 h, 40 °C), followed by signal amplification oligonucleotides (15–30 min, 40 °C), then alkaline phosphatase–conjugated Fast-Red label probe (15–30 min, 20 °C). Labeled sections were treated with chromogenic Fast-Red substrate (10 min, 20 °C), counterstained (Gill’s Hematoxylin-1/0.01% ammonia-H_2_O), mounted (Clear-Mount), and imaged under a bright-field microscope (Olympus, BX51) fitted with a digital camera (Spot RT3).

## Results

### Linkage analysis

**Figure 1:**
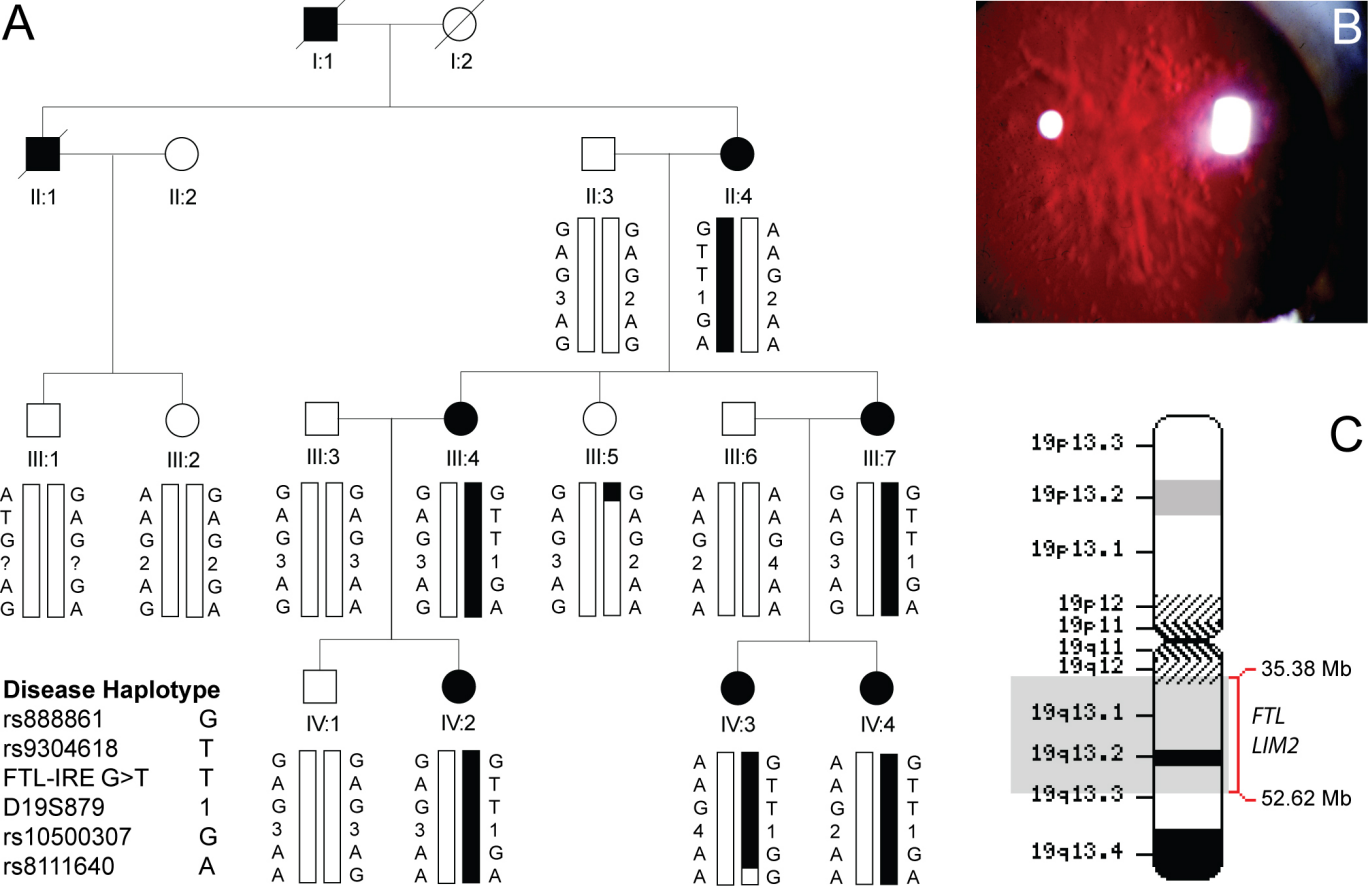
Linkage analysis of autosomal dominant cataract found to segregate in a four-generation Caucasian-American pedigree. **A**: Pedigree and haplotype analysis shows segregation of SNP and microsatellite markers on chromosome 19q, listed in descending order from the centromere (19p-tel). Squares and circles symbolize males and females, respectively. Filled symbols and bars denote affected status and haplotypes, respectively. **B**: Slit-lamp retroillumination image of a lens from individual IV:3 before surgery at ~3 months of age reveals stellate opacities that appear as paler regions of light scattering against the retinal red reflex. **C**: Ideogram of chromosome 19 indicates the location of the cataract locus (shaded gray) and two positional candidate genes.

We studied a four-generation Caucasian pedigree from the Midwestern United States, segregating star-shaped opacities affecting the lens nucleus and cortex (Figure 1). Autosomal dominant inheritance was supported by father-to-son transmission in the absence of generation skipping. Ophthalmic records indicated that the cataract was bilateral in the absence of other obvious ocular or systemic abnormalities. Age at diagnosis varied from 0.5 to 25 years, and age at surgery varied from 15 to 63 years. Postsurgical corrected visual acuity varied from 20/20 to 20/30 in the better eye.

**Figure 2:**
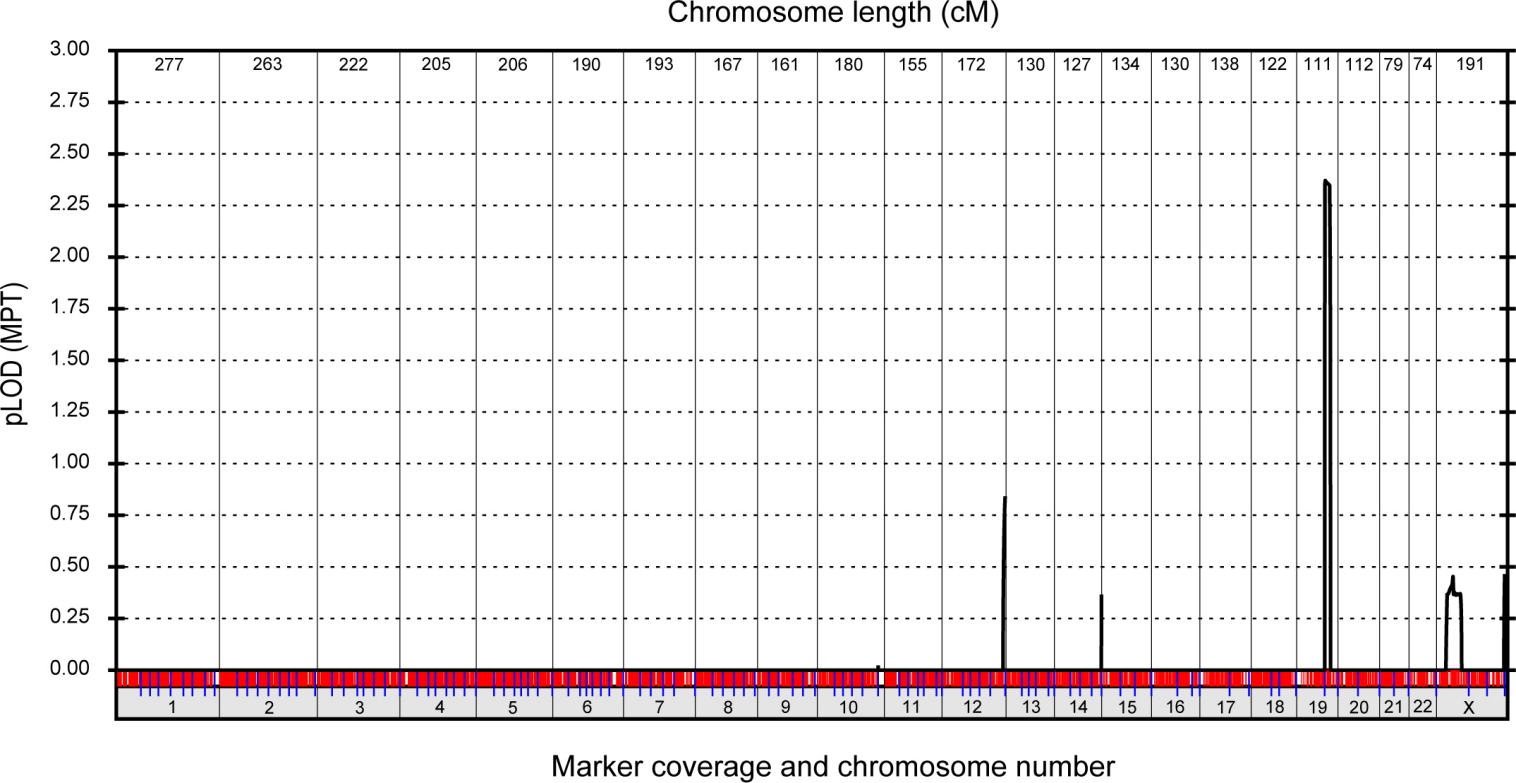
Parametric multipoint logarithm of the odds (pLOD MPT) scores were calculated for linkage between the cataract phenotype and single nucleotide polymorphism (SNP) markers across the genome. Chromosome length is shown in centi-Morgans (cM).

For genome-wide linkage analysis, the six affected and seven unaffected family members were genotyped by means of the GeneChip Mapping 10 K 2.0 Array, which comprises 10,204 SNP markers uniformly spaced at a mean intermarker distance of 258 kb, with an average heterozygosity of 0.38. Parametric multipoint analysis detected suggestive evidence of a linkage between SNP markers rs1366444 and rs2241721 (parametric LOD score 2.37) on chromosome 19q (Figure 2). Haplotype reconstruction detected two obligate recombinant individuals in the pedigree who defined a common disease interval that cosegregated with cataract in all six affected individuals (Figure 1A and Table 2). An unaffected female (III:5) was recombinant at rs888861, and an affected female (IV:3) was recombinant at rs8111640. However, no further recombinant individuals were detected at other intervening markers, consistent with the cataract locus residing in the physical interval, rs888861-(~17 Mb)-rs8111640 (chr19: 35,381,852 - 52,621,644; Figure 1C). To further validate the SNP haplotype on 19q, all 13 family members were regenotyped with microsatellite markers in the region. We obtained confirmatory evidence of linkage at marker D19S879 (Z_max_=2.03, θ_max_=0), which lies within the SNP interval (Table 2).

**Table 2:** Two-point logarithm of the odds scores (Z) were calculated for linkage between the cataract locus and markers on chromosome 19q.

Marker (recombinant)	Mb	Z at recombination fraction (Θ)=							
		0.00	0.05	0.10	0.20	0.30	0.40	Z_Max_	Θ_Max_
rs888861 (III:5)	35.38	-∞	−0.64	−0.38	−0.16	−0.06	−0.01	0.00	0.50
D19S220	38.43	1.66	1.51	1.35	1.01	0.66	0.30	1.66	0.00
rs9304618	43.47	1.77	1.63	1.49	1.15	0.77	0.37	1.77	0.00
D19S412	47.01	1.18	1.11	1.02	0.83	0.60	0.32	1.18	0.00
*FTL*-IRE c.-168G>T	49.47	2.01	1.82	1.62	1.22	0.80	0.38	2.01	0.00
D19S879	49.52	2.03	1.85	1.67	1.27	0.84	0.40	2.03	0.00
rs10500307	52.07	1.91	1.73	1.55	1.17	0.77	0.37	1.91	0.00
rs8111640 (IV:3)	52.62	-∞	−0.39	−0.15	0.04	0.08	0.06	0.08	0.31
D19S571 (IV:3)	53.30	-∞	0.79	0.91	0.82	0.57	0.25	0.91	0.11

Z values for markers on 19q are listed in physical order measured in mega base pairs (Mb) from the short-arm telomere (19ptel).

### Mutation analysis

**Figure 3:**
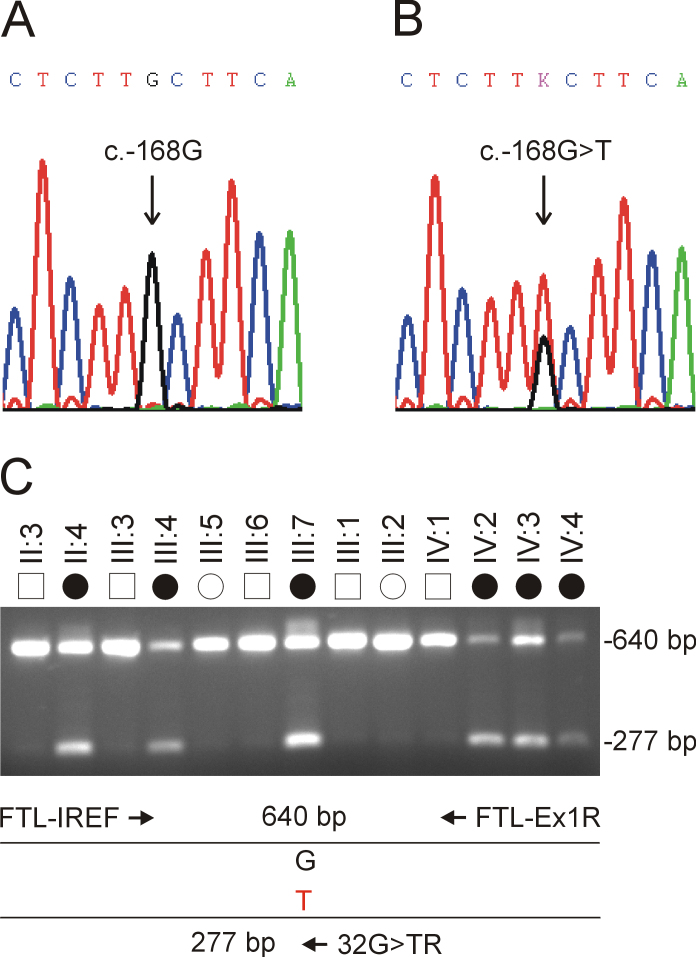
Mutation analysis was performed on the ferritin light chain – iron response element sequence. **A**: Sequence trace of the wild-type allele shows G at nucleotide 168 upstream from the ATG (c.-168G). **B**: Sequence trace of the mutant allele shows the heterozygous c.-168G>T transversion (denoted K by the International Union of Pure and Applied Chemistry code). **C**: Allele-specific PCR analysis using the three primers (Table 1) indicated by arrows in the schematic diagram, reveals that the mutant T-allele (277 bp) co-segregates with affected but not unaffected family members.

The disease interval contained over 840 positional candidate genes (NCBI Map Viewer), two of which have been associated with inherited forms of cataract. Sequencing of exons and intron boundaries first excluded the presence of coding or splice-site mutations in the gene for lens intrinsic membrane protein-2, which has been linked with autosomal recessive cataract [20,21]. However, we detected a heterozygous G-to-T transversion in exon-1 of the gene for *FTL* that was not present in the wild type (Figure 3). The change (c.-168G>T) was located in the 5′-UTR at nucleotide 168 upstream from the A (numbered +1) of the translation start-site (ATG), and within the IRE at nucleotide 32 numbered from the consensus transcription start-site (32G>T). Allele-specific PCR analysis confirmed that the T-allele cosegregated with affected, but not unaffected family members (Figure 3C). Further, when we tested the c.-168G>T transversion as a biallelic marker with a notional allelic frequency of 1% in a two-point LOD score analysis of the cataract locus (Table 2), we obtained further evidence of linkage (Z_max_=2.01, θ_max_=0). In addition, we confirmed that the c.-168G>T transversion was not listed in the NCBI SNP database (dbSNP), and excluded it as an SNP in a panel of 192 normal unrelated individuals (i.e., 384 chromosomes) using the allele-specific PCR analysis described in Figure 3C (data not shown). While it is possible that an undetected mutation lay elsewhere within the disease-haplotype interval, our genotype data strongly suggested that the c.-168G>T transversion in exon-1 of *FTL* represented a causative mutation rather than a benign SNP in linkage disequilibrium with the cataract phenotype.

### Serum ferritin and iron status

To gain a better differential diagnosis for autosomal dominant cataract cosegregating with an FTL-IRE mutation, we undertook Clinical Laboratory Improvement Amendments–certified testing of serum ferritin and iron profile in one available, affected family member (III:4). She was found to have an abnormally high serum ferritin level of 1208 µg/l (expected range 10–291 µg/l). However, her serum iron level of 66 µg/dl (range 30–160 µg/dl), total iron binding capacity value of 293 µg/dl (range 220–420 µg/dl), and calculated transferrin saturation value of 23% (range 20%–50%) were all well within the normal adult female range, and were consistent with a diagnosis of hereditary hyperferritinemia-cataract syndrome (HHCS, OMIM ID: 134790). The absence of an abnormal iron profile formally excluded the possibility of classical hereditary hemochromatosis, an autosomal recessive inborn error of iron metabolism characterized by elevated serum ferritin levels, transferrin saturation values >50%, iron overload, and variations in the hemochromatosis (HFE) gene linked to chromosome 6p [OMIM ID: 235200]. Moreover, four other genetic forms of hemochromatosis (HFE2A, HFE2B, HFE3, HFE4) were excluded, since they are associated with iron overload linked to genes other than FTL (OMIM ID: 608374, 606464, 604720, 604653).

### Iron response element variants in age-related cataract case controls

To investigate the possibility that sequence variations in the FTL-IRE region are associated with age-related cataract, we performed amplicon resequencing in 197 cases with age-related cataract and 102 age/gender-matched controls with clear lenses. While no sequence variations in the IRE of cases and controls were found (data not shown), we detected a heterozygous G-to-A transition (c.-346G>A) located upstream of the transcription start site in one case with age-related cortical cataract (Appendix 1). However, the serum ferritin levels and iron status of this individual were unknown. Similarly, a heterozygous C>A transversion located 216 nucleotides upstream of the IRE transcription start site has been reported in a cataract patient of uncertain ferritin and iron status [22]. In addition, at least 19 other variants in the immediate 5′-UTR of the FTL gene (Appendix 1) have been documented in dbSNP, one of which (rs11553230) is located within the IRE (c.-174T>C, 26T>C). However, their clinical significance is not known.

### Human ferritin light chain and mouse ferritin light chain-1 transcripts in the lens

**Figure 4:**
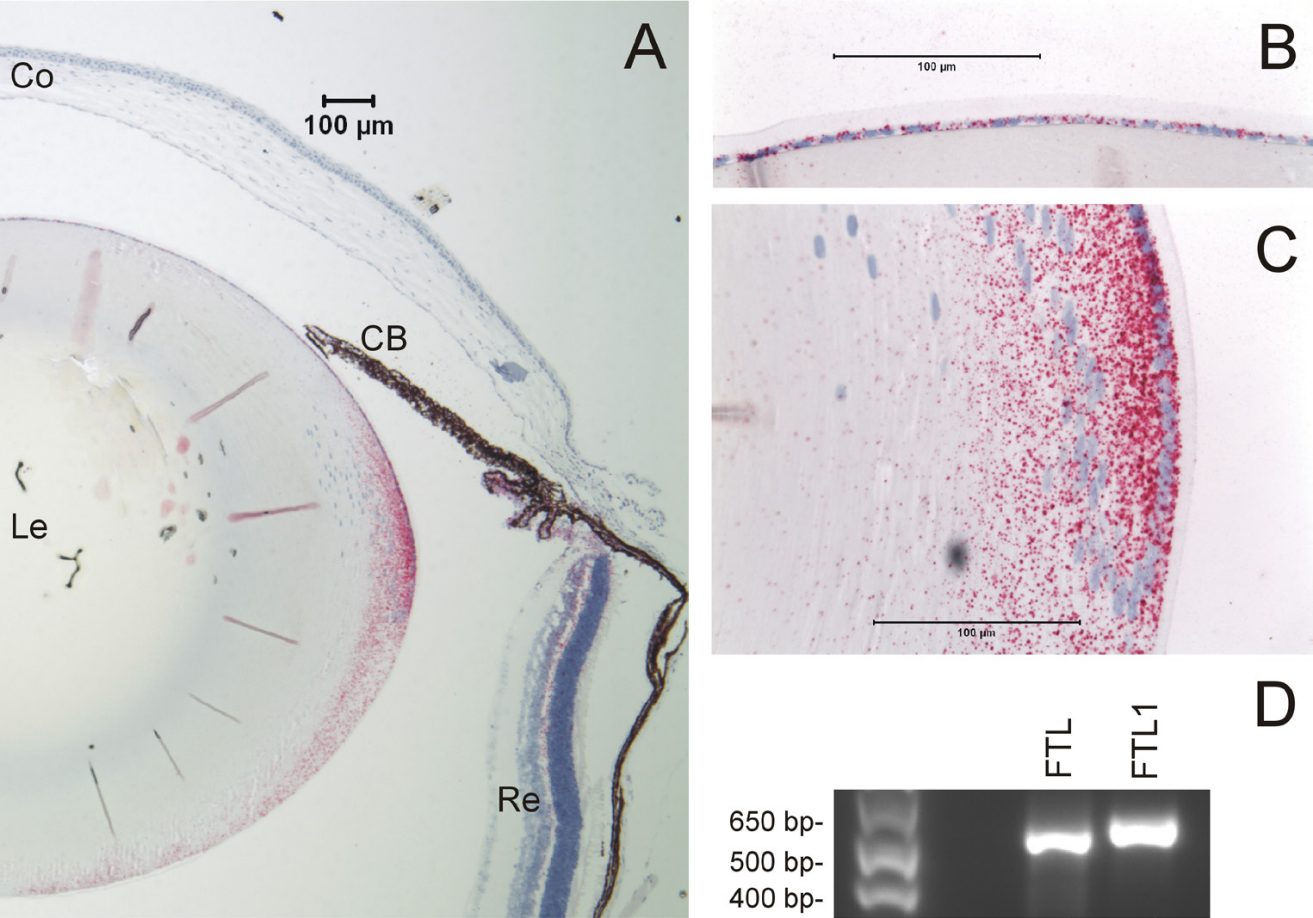
Mouse ferritin light chain-1 (FTL1) and human ferritin light chain (FTL) transcripts are expressed in the lens. **A**: Low magnification image (sagittal plane) of the mouse eye (postnatal day 21) shows strong localization of ferritin light chain-1 (FTL1) transcripts (red punctate staining) to the equatorial epithelium and peripheral cortical fiber cells of the lens (Co - cornea, CB - ciliary body, and iris, Le - lens, Re - retina). **B** and **C**: High-magnification images of FTL1 transcripts expressed in the lens anterior epithelium (**B**) and equatorial “bow” region (**C**). Scale bars represent 100 µm. Cell nuclei are stained blue. **D**: Reverse-transcription polymerase chain reaction (RT–PCR) analysis of lens RNA detects amplicons containing complete coding sequence for FTL and FTL1. Note the FTL1 amplicon (184 codons) is expected to be slightly larger than that of FTL (176 codons).

To determine the expression profile of human FTL and its mouse counterpart, FTL1, in the lens we undertook RT–PCR amplification and ISH analyses. RT–PCR confirmed that the full-length coding regions for human FTL (codons 1–175 + stop) and mouse FTL1 (codons 1–183 + stop) could be amplified from human and mouse lenses, respectively (Figure 4D). ISH revealed that FTL1 messenger RNA (mRNA) transcripts are most strongly expressed in the equatorial epithelium and peripheral cortical fiber cells of the young (postnatal day 21) mouse lens, with much lower levels detected in other eye tissues (Figure 4). This preferred expression profile of the lens is consistent with FTL1 transcript levels detected by microarray analysis of mouse eye tissues [23].

## Discussion

In this study, we have identified a Caucasian-American family segregating HHCS. Since it was first described in 1995 [24-27], HHCS has been reported in over 70 families or individuals mostly of European descent, with a minimum estimated prevalence of 1/200,000 [28]. Mutations underlying HHCS are all clustered in the IRE of the FTL gene (Appendix 1 and Appendix 2). This motif comprises approximately 75 nucleotides of the 5′-UTR, that when transcribed into mRNA, folds into a hairpin-like secondary structure with a characteristic hexanucleotide loop joining the 5′- and 3′-stem regions generated by Watson–Crick base pairing [29,30]. The FTL-IRE mutation spectrum comprises at least 34 heterozygous mutations, including 27 single nucleotide or point changes, two compound heterozygous changes, and five deletions ranging from 2 to 29 bp (Appendix 1 and Appendix 2). The most prevalent mutations occur in the 5′-stem and hexanucleotide loop regions. The c.-168G>T (32G>T) change described here is located in the 5′-stem region, adjacent to the unpaired cytosine “bulge” (c.-167C, 33C), and recurrent variations at this site have been reported in at least 17 other families of European ancestry and one of Indian descent, making it one of the most prevalent mutation sites in the IRE [28,31-36]. Outside of the IRE, rare mutations in the coding region of FTL have been associated with either neuroferritinopathy or hyperglycosylated serum ferritin associated with benign hyperferritinemia in the absence of cataract [37-40].

HHCS has been shown to result from disruption of the iron-dependent translational control of FTL gene expression. Specifically, HHCS mutations have been shown to alter the secondary structure and thermodynamic stability of the FTL-IRE stem-loop structure, and to reduce its binding affinity for cytoplasmic iron regulatory proteins (IRP1, IRP2) that actively repress FTL translation [29,41,42]. Notably, molecular modeling predicts that the FTL-IRE mutation found here disrupts base-pairing between c.-168G (32G) in the 5′-stem and c.-150C (50C) in the 3′-stem, inducing altered base pairing within the upper stem and hexaloop regions [32]. Further, protein binding assays have confirmed that the same c.-168G>T mutation significantly reduced IRP binding affinity in vitro [29,31]. Such mutation-induced loss of IRE-IRP interaction is expected to result in elevated levels of FTL biosynthesis, and large crystalline deposits of immunoreactive L-ferritin have been observed in lens tissue recovered from HHCS patients following cataract surgery [32,42,43]. Our expression data show that FTL transcription is higher in the lens compared with other eye tissues (Figure 4), and further support the concept that even in the presence of a wild-type FTL-IRE allele, a mutant FTL-IRE allele causes autosomal dominant cataract through uncontrolled (derepressed) translation of L-ferritin.

While HHCS is generally associated with childhood onset of cataract, there is considerable variation in the appearance and progression of lens opacities (Appendix 2), and many patients do not undergo cataract surgery until middle age and beyond. Such observations raise the possibility that FTL-IRE sequence variations may also contribute to the etiology of age-related cataract. Our sequencing data excluded IRE variations in a European cohort with clinically well-defined age-related cortical, nuclear, and posterior subcapsular lens opacities. A similar study also failed to detect evidence for acquired somatic mutations in the FTL-IRE sequence of DNA extracted from lens capsular material recovered following age-related cataract surgery in an Israeli cohort [34]. In contrast, we detected a heterozygous G>A transition (c.-346G>A) located 147 nucleotides upstream of the IRE that was not listed in the SNP databases in a patient with age-related cortical cataract but unknown serum ferritin or iron status. Another variation (C>A) located 216 nucleotides upstream of the IRE transcription start site has been associated with elevated levels of FTL mRNA in lymphocytes from a cataract patient also of undisclosed serum ferritin and iron status [22]. In summary, while FTL-IRE variations clearly underlie HHCS, they do not appear to be frequently associated with age-related cataract in Europeans. Further studies will be required to determine the significance of FTL 5′-UTR and promoter variations located outside the IRE in the genetic etiology of age-related cataract.

## Appendix 1. Genetic variation in the iron response element (IRE) and immediate flanking 5´-untranslated region (5´-UTR) of the ferritin light chain (FTL) gene.

**A:** Schematic of FTL-IRE sequence (shaded) showing location of point-mutations (red), deletions >6bp (bold), deletions 2–6bp (bold-underline), and 19 variants, listed in dbSNP (green). The IRE sequence spans ~75 nucleotides between −125c and −199 g, and forms a hairpin-like structure in transcribed mRNA with 5′- and 3′- stem regions (gray), a hexa-nucleotide loop (yellow), and unpaired 5′-stem regions (cyan). The first base, A, of the translation start-site (ATG) is numbered +1. The transcription start-site is located at nucleotide −199 g and is numbered 1 in blue. Ex1->=start of exon-1 at nucleotide −282a. **B:** Sequence alignment of human (Hs) versus mouse (Mm) IRE showing six mismatches (red) in the 5′- and 3′- stem regions but not in the loop region (yellow). To access the data, click or select the words “Appendix 1.”

## Appendix 2. Mutation spectrum of the ferritin light chain-iron response element (FTL-IRE) sequence on 19q13.4.

To access the data, click or select the words “Appendix 2.”
